# Induction of telomere shortening and cellular apoptosis by sodium meta-arsenite in human cancer cell lines

**DOI:** 10.1080/19768354.2017.1342691

**Published:** 2017-07-01

**Authors:** Yoon-Dong Kim, Si-Jeong Jang, Eun-Ji Lim, Jeong-Sook Ha, Sharath Belame Shivakumar, Gie-Joon Jeong, Gyu-Jin Rho, Byeong-Gyun Jeon

**Affiliations:** a Department of Biology Education, College of Education, Gyeongsang National University, Jinju, Republic of Korea; b OBS/Theriogenology and Biotechnology, College of Veterinary Medicine, Gyeongsang National University, Gyeongsangnam-do, Republic of Korea; c Research Institute of Education, Gyeongsang National University, Jinju, Republic of Korea

**Keywords:** Human, cancer, sodium meta-arsenite, telomere length, apoptosis

## Abstract

The present study assessed the cytotoxicity of sodium meta-arsenite (SMA) on telomere shortening and cellular apoptosis in human A-549, MDA-MB-231 and U87-MG cancer cell lines. Following 2 weeks of 1 μM SMA treatment, population doubling time (PDT) was significantly (*P* < .05) increased by the inhibition of cell proliferation in all the cancer cell lines compared to that in untreated controls. Level of telomerase activity by relative-quantitative telomerase repeat amplification protocol was significantly (*P* < .05) downregulated by SMA treatment with significant (*P* < .05) decrease of both telomerase reverse transcriptase and telomerase RNA component transcripts, responsible for telomerase activity. A significant (*P* < .05) shortening of telomeric repeats by telomere restriction fragment analysis was consequently observed in SMA-treated cells. Moreover, high incidence of cells with senescence-associated β-glucosidase activity was observed in SMA-treated cells and some cells were also differentiated into adipocytes probably due to the loss of tumorous characterizations. Cellular apoptosis proven by DNA fragmentation was observed, and intrinsic apoptotic transcripts (BAX, caspase 3 and caspase 9) and stress-related transcripts (p21, HSP70 and HSP90) were significantly (*P* < .05) increased in three cancer cell lines treated with SMA. Based on the present study, SMA treatment apparently induced a shortening of telomere length and cytotoxicity, such as induction of cell senescence, apoptosis and cell differentiation. Therefore, we conclude that SMA treatment at specific concentration can lead to gradual loss of tumorous characterizations and can be considered as a potential anti-cancer drug for chemotherapy treatment.

## Introduction

Malignant tumors or cancers are characterized by their unlimited as well as higher proliferation capacity with uncontrolled cell cycle properties. The growing cancer cells can easily metastasize into other tissues of the body. The cell division and proliferation in most of the cells are generally controlled by various types of molecules, including survival factors, mitogens, growth factors and others that promote cell proliferation. However, most of the cancer cells exhibit unlimited cell proliferation capacity under chemically defined conditions even after starving cell-division-promoting molecules (Rozengurt 1983). Besides maintaining an unlimited cell proliferation capacity, cancer cells are distinctly associated with the maintenance of telomeric repeats. Telomeric repeats are a variable number of specialized and repeated simple DNA sequences (TTAGGC) capped at the ends of linear chromosomes of eukaryotes including humans (Cong et al. 2002; Arnoult and Karlseder 2015). In most of the normal somatic cells, the linear chromosomes exhibit serious structural problems and cannot extend the DNA strand, while the last piece of RNA primer on the lagging strand is removed for DNA replication. The end-replication problem induced during DNA replication leads to shortening of telomeric repeats. Thus, the telomeric repeats in the normal somatic cells are gradually shortened through each cell division by the end-replication problem (Allsopp et al. 1992; Aubert and Lansdorp 2008; Arnoult and Karlseder 2015). The cells with uncapped or fully exhausted telomeric repeats arrest their cell cycle and finally enter a senescent stage and/or programmed cell death. Hence, the telomeric repeats play a central role in genetic stability and protects the end of chromosomes (Aubert and Lansdorp 2008; Arnoult and Karlseder 2015). Therefore, the normal somatic cells possess the capacity of limited cell division by shortening of telomeric repeats (Allsopp et al. 1992; Levy et al. 1992; Blasco et al. 1997; Capper et al. 2007; O’Sullivan and Karlseder 2010). On the other hand, several types of cells such as embryos, embryonic stem cells and most cancer cells retain their unlimited cell division capacity with their telomeric repeats continually stabilized without undergoing shortening. The maintenance of telomeric repeats is undoubtedly related with higher levels of telomerase activity. Telomerase is an enzyme called reverse transcriptase, which adds the telomeric repeats using telomere RNA to the end of their chromosome. Higher levels of telomerase activity has been observed in immortalized cells, including cancer cells (Aubert and Lansdorp 2008; Artandi and DePinho 2010; Arnoult and Karlseder 2015). However, telomerase activity is found to be very low or undetected in normal cells, displaying shortening of telomeric repeats, and eventual cellular apoptosis (Shay et al. 2001; Blasco 2005).

The upregulation of telomerase activity is a representative hallmark of cancerous cells showing capacity of unlimited cell proliferation (Blasco 2005). It has been reported that the induction of cellular senescence and apoptosis through shortening of telomeric repeats is very difficult in immortalized cancer cells having increased telomerase activity. Accordingly, anti-cancer drugs or compounds, including 6-thio-2′-deoxyguanosine, BRACO-19, sodium meta-arsenite (SMA) and others, have been consistently developed to decrease the telomerase activity and/or shortening of telomeric repeats (Burger et al. 2005; Jeon et al. 2011a; Maida and Masutomi 2015; Mender et al. 2015; Xu and Goldkorn 2016). Previous studies have particularly demonstrated that SMA can induce the cytotoxicity, such as inhibition of cell proliferation in various types of normal and cancer cells (Phatak et al. 2008; Ruiz-Ramos et al. 2009; Jeon et al. 2011a; Woo et al. 2014). Besides, it has been suggested that the anti-cancer effect of the SMA is obviously combined with telomeric repeats at a ratio of one molecule per three TTAGGG telomeric repeats and the combination can be induced to shortening and erosion of the telomeric repeats by reactive oxygen species (ROS) production. And the reports have thus suggested that the erosion effect of telomeric repeat is more predominant in the cancer cell lines with short length of telomeric repeats than those with long length of telomeric repeats (Phatak et al. 2008). Previously we have demonstrated that the cytotoxicity of SMA is tightly directly associated with the length of telomeric repeats, and more effective cytotoxicity with lower IC_50_ values is exhibited in the cancer cell lines, such as A-549, MDA-MB-231, MCF-7 and U87-MG with shortened telomere length, compared with normal cell lines with relatively long telomere length, such as normal muscle cells and mesenchymal stem cells derived from adult dental tissues (Jeon et al. 2011a). Thus, SMA can be regarded as a potent and selective anti-cancer drug for chemotherapy or alternative treatment by inducing erosion of telomeric repeats of the cancer cells. However, the cytotoxic effect on the shortening of telomeric repeats is not yet investigated in the cancer cells treated with SMA during prolonged culture time.

In the present study, we preferentially investigated the effect on the shortening of telomeric repeats and telomerase activity in the human A-549 lung adenocarcinoma, MDA-MB-231 breast adenocarcinoma and U87-MG brain glioblastoma astrocytoma cells treated with 1 μM SMA during prolonged culture time of up to 2 weeks. Besides, the incidence of cellular senescence and apoptosis was further examined in each of the cancer cell lines treated with SMA.

## Materials and methods

### Culture and treatment of cells

All chemicals and media used in this study were purchased from both Sigma (USA) and Invitrogen (USA), unless otherwise specified. A-549 lung adenocarcinoma, MDA-MB-231 breast Adenocarcinoma, U87-MG brain glioblastoma astrocytoma cancer cell lines and MRC-5 normal fetal lung fibroblasts were supplied from the American Type Culture Collection (USA). The complete cell culture medium for cancer cell lines was composed of advanced-Dulbecco’s modified eagle medium (A-DMEM) supplemented with 3% fetal bovine serum (FBS) and 1.0% penicillin-streptomycin (10,000 IU and 10,000 μg/ml, respectively) at 37.5°C in a humidified atmosphere of 5% CO_2_ in air. Otherwise, MRC-5 fibroblasts were maintained in the A-DMEM-supplemented 10% FBS. On reaching 80–90% confluent status, the cells were continually sub-cultured and the culture medium was changed twice a week. For the SMA treatment, the cancer cell lines were exposed in A-DMEM containing 0 (untreated control) and 1 μM SMA for up to 2 weeks. The treated cells were detached with 0.25% trypsin-EDTA solution and the cell pellets were stored at −80°C for further analysis.

### Analysis of population doubling time (PDT)

Cells from each cancer cell line were seeded at 0.5 × 10^4^ cells/well into a 6-well plate separately and cultured in complete A-DMEM media containing 0 (untreated control) and 1 μM SMA for up to 2 weeks at 37.5°C in a humidified atmosphere of 5% CO_2_ in air, and the culture medium was changed twice a week. After 2 weeks, the treated cells were harvested with 0.1% (w/v) trypsin and counted using a hemocytometer. The PDT value was calculated using the formula DT = *t*(log 2)/(log Nt−log No), where *t* is the culture time, and No and Nt are the initial and final cell numbers after seeding, respectively.

### Analysis of telomerase activity by relative-quantitative telomerase repeat amplification protocol

The cells from each cancer cell line were seeded at 1 × 10^5^ cells into a T-75 cell culture flask and cultured in complete A-DMEM media containing 0 (untreated control) and 1 μM SMA. After culturing for up to 2 weeks, the cells were dissociated by 0.1% (w/v) trypsin treatment and either immediately analyzed or further frozen at −80°C for measurement of telomerase activity and telomere length. For detecting telomerase activity, the conventional PCR-based assay protocol was revised into the relative-quantitative telomeric repeat amplification protocol (RQ-TRAP) using Rotor Gene Q (Qiagen, USA), real-time PCR machine, as previously described by Jeon et al. (2011b). Briefly, the control and SMA-treated cells were harvested at 1 × 10^5^ cells per sample. Each of the samples was lysed with 400 µl of 0.5% (v/v) 1·3-[(3-cholamidopropyl) dimethylam-monio] propanesulfonic acid (CHAPS) lysis buffer (pH 7.5) supplemented with 10 mM Tris-HCl, 1 mM MgCl_2_, 1 mM EGTA, 0.1 mM benzamidine, 5 mM β-mercaptoethanol and 10% glycerol for 30 min at 4°C, and subsequently centrifuged for 20 min at 12,000×*g* at 4°C. An 80% volume of supernatant from each of the lysed samples was transferred to a fresh new sample tube and the concentration of total protein was measured with a spectrophotometer (Mecasys, Korea). The reaction mixture for RQ-TRAP was composed of Rotor-Gene™ 2× SYBR Green (Qiagen, USA), 5 μg total protein of each of the lysed sample, 2.5 mM MgCl_2_, 0.02 µg of telomerase TS primer and 0.04 µg of anchored return ACX primer which are shown in Table 1. The final volume of the reaction mixtures was adjusted into 20 µl with RNase-free water. The reaction mixtures were precedently processed for 30 min incubation at 30°C and 10 min at 94°C to denature each of the samples. And all of the samples were subsequently amplified in 40 PCR cycles comprising 94°C for 30 s, 60°C for 90 s and 72°C for 0 s. The relative quantification of all the samples was calculated with the second derivative method of crossing point (Cp) determination using Gene Q Series Software (Qiagen, USA). The relative level of telomerase activity in untreated control and 1 μM SMA-treated sample was calculated, based on the level of telomerase activity considered as 100% in untreated MRC-5 fibroblasts, and at least five replicates of RQ-TRAP were carried out in each sample.

**Table 1.** Primer sequences, PCR product size and annealing temperature used for RQ-TRAP and RT-PCR.

Gene	Primer sequences (5′–3′)	Amplification size (bp)	Annealing temp (°C)
RQ-TRAP TS	AATCCGTCGGAGCAGAGTT		60
RQ-TRAP ACX	GCGCGGCTTACCCTTACCCTTACCCTAACC		60
GAPDH	GAAGGTGAAGGTCGGAGTC GAAGATGGTGATGGGATTTC	228	57
TERT	CGGAAGAGTGTCTGGAGCAA GGATGAAGCGGAGTCTGGA;	198	60
TERC	TCTAACCCTAACTGAGAAGGGCGTAG, GTTTGCTCTAGAATGAACGGTGGAAG	126	60
BAX	TCTGACGGCAACTTCAACTG AGTCCAATGTCCAGCCCATG	127	60
Caspase-3	TGAGCCATGGTGAAGAAGGA TCGGCCTCCACTGGTATTTT	220	55
Caspase-9	CTCTTGAGAGTTTGAGGGGAAA ACTCACGGCAGAAGTTCACA	105	55
p21	TGGCAGTAGAGGCTATGGA AACAGTCCAGGCCAGTATG	178	57
HSP70	ACGAATCCCTGCGGTAAAAG AAAGCAGCGATAAGATGGC	127	60
HSP90	ACAAGCACATATGGCTGGAC TCTTTGCTGCCATGTAACCC	94	58

### Analysis of telomere length by chemiluminescent assay

After in vitro cell culture for up to 2 weeks in complete A-DMEM media containing 0 (untreated control) and 1 μM SMA, the telomere length of cancer cells from different cell lines was analyzed by non-radioactive chemiluminescent assay protocol with TeloTAGGG telomere restriction fragment length assay kit (Roche, USA), according to the manufacturer’s instructions. Briefly, the genomic DNA in the untreated control and SMA-treated cells was extracted with total DNA purification kit (GeneAll, Korea). Following measurement of the extracted DNA concentration with a spectrophotometer (Mecasys, Korea), 1 μg of total DNA was digested in the buffer containing a mixture of Hinf I and Rsa I restriction enzymes for 2 h at 37°C. The DNA fragments were run in 0.8% agarose gel, subsequently treated with HCl, denaturation buffer and neutralization buffer. The treated gel was transferred onto a positively charged nylon membrane (Roche, USA). The membrane was treated with a digoxigenin (DIG)-labeled telomere hybridization probe (Roche, USA) at 42°C for 3 h, washed with high stringency buffer and then treated with anti-DIG-alkaline-phosphatase buffer for 30 min. After being rinsed with washing buffer, the membrane was exposed to X-ray film for 20–30 min at 25°C. The images of the telomeric repeats on the X-ray film were acquired by an image scanning system. The length of telomeric repeats was determined at a spot with the highest intensity using Gelviewer image-processing software (Innogene, Korea).

### Analysis of senescence-associated β-galactosidase activity

The cellular frequency of the cells with activity of senescence-associated β-galactosidase was investigated in the 0 (untreated control) and 1 μM SMA-treated cells using cell senescence assay kit (Cell Signaling Technology, USA), according to the manufacturer’s protocols. Briefly, each type of cancer cells were seeded at 0.5 × 10^4^ cells/well into a 6-well plate, and cultured in complete A-DMEM media containing 0 and 1 μM SMA for up to 2 weeks. After being washed with D-PBS, the cells were treated in 1 ml of fixative solution contained in the kit for 10–15 min at room temperature. The cells were then subsequently incubated with 1 ml of senescence-associated β-galactosidase staining solution at 37°C for overnight. The treated cells were examined under an inverted microscope (Nikon, Japan) equipped with CCD camera and image program. The cells stained with blue color were considered as positive for senescence.

### Analysis of DNA fragmentation by Wright-Giemsa staining

The DNA fragment displayed in the apoptotic cells was also investigated by Wright-Giemsa staining assay in the 0 (untreated control) and 1 μM SMA-treated cells. Briefly, cells of each cancer cell line were seeded at 0.5 × 10^4^ cells/well into a 6-well plate, and cultured in complete A-DMEM media containing 0 and 1 μM SMA for up to 2 weeks. After being cultured for 2 weeks, the cells at mitotic status were removed by washing with D-PBS and shaking culture flask. The attached remnant cells were fixed in methanol for 2 min, and stained with Wright-Giemsa staining solution for 15 min. After removing the staining solution, the cells were treated with 10% Wright-Giemsa buffer solution (in PBS) for 5 min and again washed with D-PBS. The cells with fragmented DNA were examined under an inverted microscope (Nikon, Japan) equipped with an image system. Further, the lipid-like droplets were frequently observed in the three cancer cell lines treated with 1 μM SMA. And the cells were fixed with 3.7% paraformaldehyde overnight to investigate cellular differentiation into the adipocyte and stained with 0.5% Oil Red O kit, adipocyte-specific staining solution, for 2 h at room temperature and were also examined under an inverted microscope (Nikon, Japan).

### Quantitative analysis of transcripts by RT-PCR

The expression level of transcripts associated with telomerase and apoptosis was investigated in 0 (untreated control) and 1 μM SMA-treated cells. The isolation and purification of total RNA from cells was carried out using an RNeasy Micro Kit (Qiagen, USA), according to the manufacturer’s protocols with minor modification of DNase treatment to remove contaminated genomic DNA. The concentration of extracted total RNA was measured with a spectrophotometer (Mecasys, Korea). One microgram of total RNA from each sample was synthesized into cDNA using Omniscript reverse transcription kit (Qiagen, USA). The reaction samples, including 2 μl of 10 μM random hexamer (Invitrogen, USA), 1 μl of 10 U/μl RNase inhibitor (Invitrogen, USA), 2 μl RT buffer, 2 μl dNTP and 1 μl Omniscript (Qiagen, USA), were finally adjusted to a total volume of 20 μl with RNase-free water, and treated in a thermal cycler PCR machine (TaKaRa, Japan) for 1 h at 42°C, followed by additional 5 min at 95°C for inactivation of the reverse transcriptase. At least three cDNA reactions were converted from each of the RNA samples. Each of the cDNA samples was amplified out in a Rotor Gene Q (Qiagen, USA), real-time PCR machine. Each PCR tube contained 2 μl of Rotor-Gene™ 2× SYBR Green (Qiagen, USA), 2 μl of cDNA template and each 1 μl of primer (forward and reverse, 10 μM) in 20 μl of reaction volume. The amplification protocol was composed of denaturation for 15 s at 95°C, annealing for 10 s at 58–60°C and extension for 16 s at 72°C in 30 cycles and quantification of PCR was estimated by analysis of threshold value (Ct value) with Rotor Gene Q software (Qiagen, USA). The expression level of each transcript in all samples was calculated, based on the level of reference gene, GAPDH. If necessary, the size and intensity of amplified PCR products were investigated on the 1% agarose gel with reaction mixtures amplified by the real-time PCR machine. The details of primers are provided in Table 1.

### Statistical analysis

Differences among cell groups were analyzed by using one-way analysis of variance (ANOVA, SPSS 15.0, USA). All data were expressed as mean ± standard error. The differences were considered significant when *P* < .05.

## Results

### Analysis of PDT

**Figure 1.**
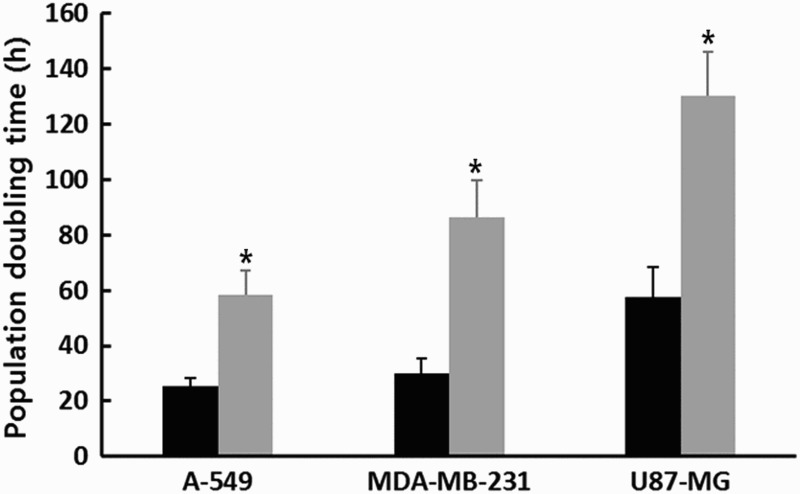
Change in PDT in untreated control (▪) and 1 μM SMA-treated (▪) A-549, MDA-MB-231 and U87-MG cancer cells. Asterisks (*) indicates significant (*P* < .05) difference between untreated control and SMA-treated cell lines, respectively.

Following 1 μM SMA treatment for up to 2 weeks, the PDT) was analyzed by counting cell numbers, as shown in Figure 1. The PDT was 25.4 ± 2.78, 30.1 ± 5.32 and 57.7 ± 10.68 h in the untreated control A-549, MDA-MB-231 and U87-MG cancer cell lines in three replicates, respectively. The A-549 and MDA-MB-231 cancer cells generally displayed a higher proliferation rate compared with that of U87-MG. However, the PDT was 58.5 ± 8.67, 86.5 ± 13.45 and 130.5 ± 15.72 h in the A-549, MDA-MB-231 and U87-MG cancer cell lines treated with 1 μM SMA, respectively. The mean PDT was significantly (*P* < .05) increased by 1 μM SMA treatment.

### Analysis of telomerase activity

**Figure 2.**
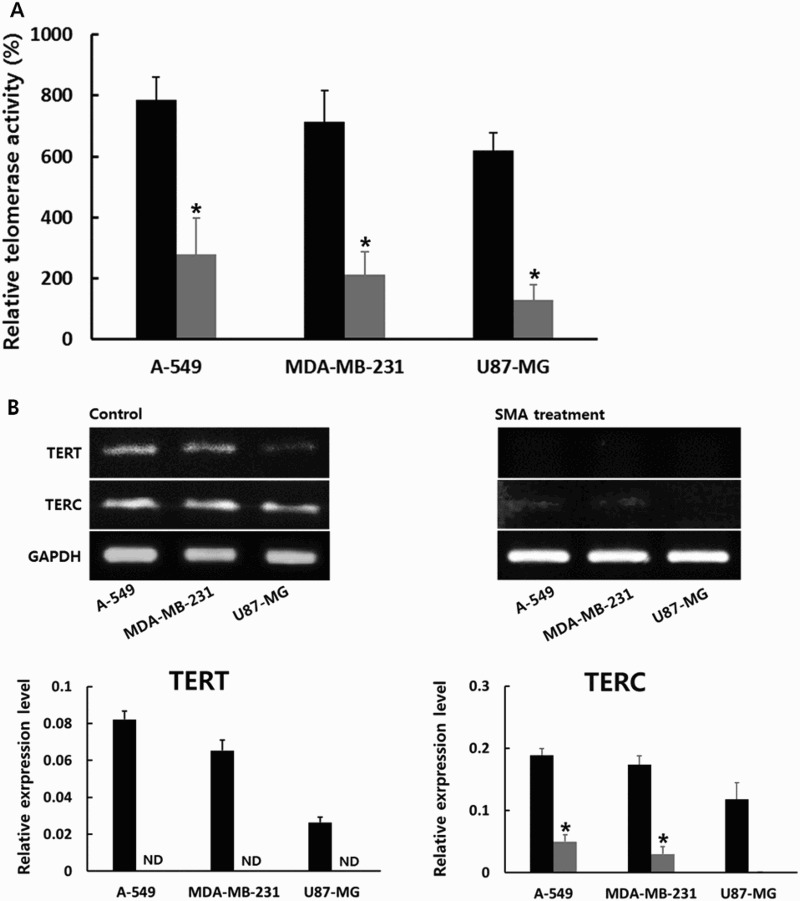
A, Level of telomerase activity analyzed by RQ-TRAP assay in untreated control (▪) and 1 μM SMA-treated (▪) A-549, MDA-MB-231 and U87-MG cancer cells. B, Expression level of TERT and TERC transcripts related with telomerase activity in untreated control (▪) and 1 μM SMA-treated (▪) A-549, MDA-MB-231 and U87-MG cancer cells. Asterisks (*) indicates significant (*P* < .05) difference between untreated control and SMA-treated cell lines, respectively.

The RQ-TRAP assay using real-time PCR machine was employed to examine the level of telomerase activity in A-549, MDA-MB-231 and U87-MG cancer cells treated with 1 μM SAM for up to 2 weeks. As shown in Figure 2(A), the level of telomerase activity in untreated normal MRC-5 fibroblasts was considered as 100% and the relative level of telomerase activity in each cancer cell line was determined on the basis of this level. The relative level of telomere activity was 785 ± 77.3, 714 ± 103.2 and 619 ± 58.9% in the untreated A-549, MDA-MB-231 and U87-MG cancer cells, respectively. The high level of telomerase activity was exhibited by all the cancer cell lines compared with that of normal MRC-5 fibroblasts. However, the relative level of telomerase activity was 278 ± 112.3, 212 ± 75.1 and 129 ± 50.5% in the A-549, MDA-MB-231 and U87-MG cancer cells treated with 1 μM SMA, respectively. The relative level of telomerase activity was significantly (*P* < .05) downregulated by 1 μM SMA exposure for up to 2 weeks. Especially, the telomerase activity in U87-MG cancer cells treated with 1 μM SMA almost reached the level as that in MRC-5 fibroblasts.

Furthermore, the expression level of telomerase reverse transcriptase (TERT) and telomerase RNA component (TERC) transcripts, closely related with telomerase activity, was investigated by RT-PCR assay in the A-549, MDA-MB-231 and U87-MG cancer cells treated with 1 μM SMA. The result is shown in Figure 2(B). The relative expression level was quantified by analysis of Ct value based on the level of GAPDH. The relative expression level of TERT and TREC transcripts was 8.2 ± 0.45 and 18.8 ± 4.79; 6.5 ± 0.56 and 17.3 ± 2.99; and 2.6 ± 0.29 and 11.8 ± 6.67% in the untreated A-549, MDA-MB-231 and U87-MG cancer cells, respectively. The TERT and TERC transcripts were highly expressed in A-549 and MDA-MB-231 cancer cells than in U87-MG cancer cells. However, the expression of TERT was not detected in all types of cancer cells after 1 μM SMA treatment, and the relative expression level of TREC transcript was 4.9 ± 1.08, 1.42 ± 1.21 and 0% in the A-549, MDA-MB-231 and U87-MG cancer cells treated with 1 μM SMA, respectively. One micrometer SMA exposure for up to 2 weeks induced significantly (*P* < .05) decreased expression level of TERT and TERC transcripts in each cancer cell line.

### Analysis of telomere length

**Figure 3.**
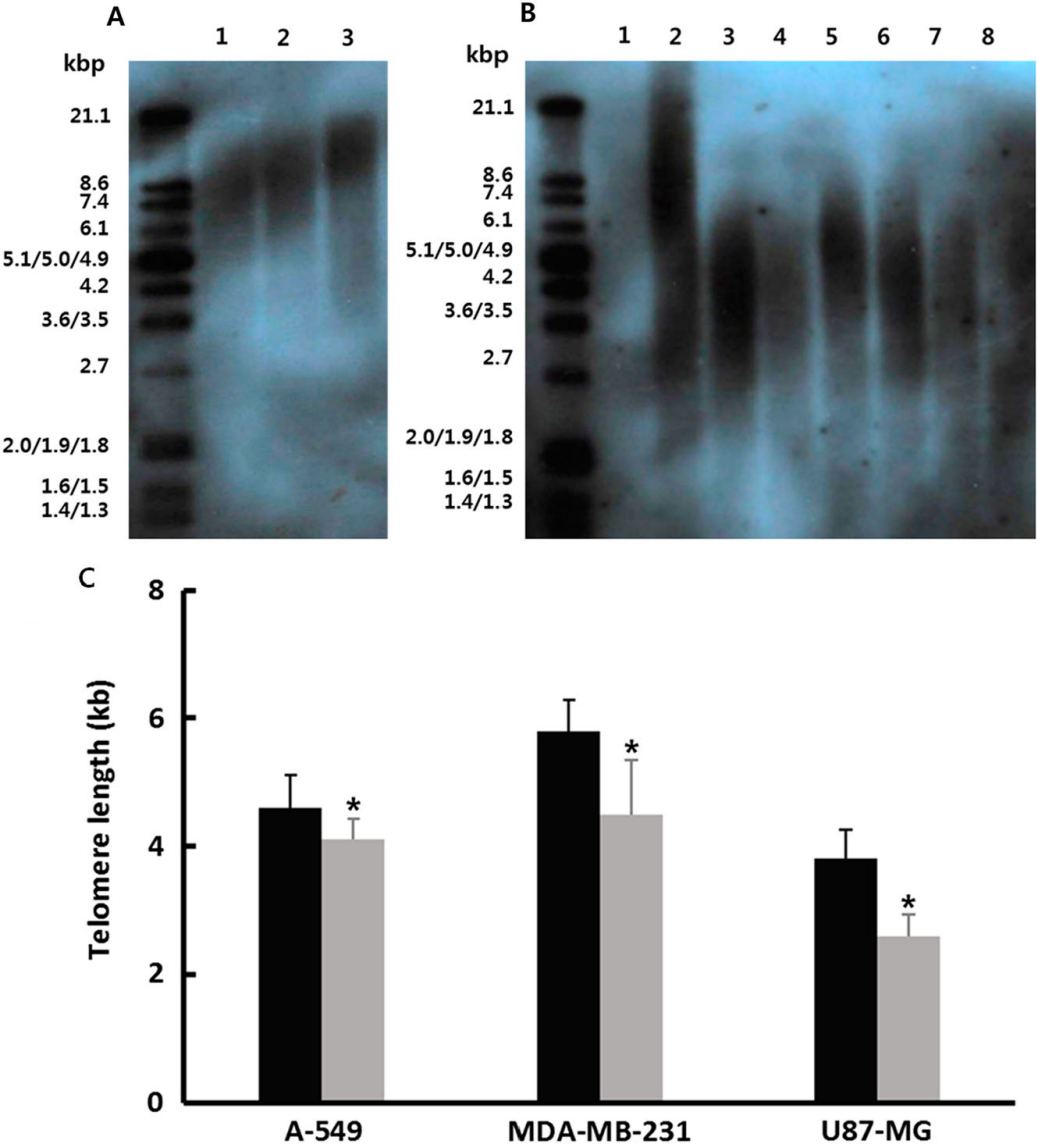
A, Telomere length in untreated control and 1 μM SMA-treated A-549, MDA-MB-231 and U87-MG cancer cells. A, Range of telomere length in normal fibroblasts as control cells. Lane 1: passage 15; Lane 2: passage 10; Lane 3: passage 5. B, Mean (±SEM) Telomere length in 1 μM SMA-treated A-549, MDA-MB-231 and U87-MG cancer cells. Lane 1: Negative control; Lane 2: positive control with mean 10.2 kbp telomere length. Lane 3: untreated A-549 cells; Lane 4: 1 μM SMA-treated A-549 cells; Lane 5: untreated MDA-MB-231 cells; Lane 6: 1 μM SMA-treated MDA-MB-231 cells; Lane 7: untreated U87-MG cells; Lane 8: 1 μM SMA-treated A U87-MG cells. C, Mean (±SEM) telomere length in untreated control (▪) and 1 μM SMA-treated (▪) A-549, MDA-MB-231 and U87-MG cancer cells with three replicates. Asterisks (*) indicates significant (*P* < .05) difference between untreated control and SMA-treated cell lines, respectively.

The non-radioactive chemiluminescent assay was employed for measuring telomere length in A-549, MDA-MB-231 and U87-MG cancer cells treated with 1 μM SMA exposure for up to 2 weeks. The result is shown in Figure 3(B). As control, the telomere length was preferentially measured in the normal MRC-5 fibroblasts at passages 5, 10 and 15, respectively (Figure 3(A)) and progressive shortening of the telomere length was observed during prolonged culture time. The telomere length was 4.6 ± 0.51, 5.8 ± 0.49 and 3.8 ± 0.47 kilo base pairs (kbp) at spot with the highest density on the transferred membrane in the untreated control A-549, MDA-MB-231 and U87-MG cancer cells, respectively. The mean length of telomere in cancer cell lines was shorter than that of normal MRC-5 fibroblasts. However, the telomere length was 3.9 ± 0.32, 4.5 ± 0.84 and 2.6 ± 0.33 kbp in the A-549, MDA-MB-231 and U87-MG cancer cells treated with 1 μM SMA, respectively (Figure 3(C)). The length of telomeric repeats was significantly (*P* < .05) decreased by 1 μM SMA treatment for up to 2 weeks.

### Analysis of senescence-associated-β-galactosidase activity

**Figure 4.**
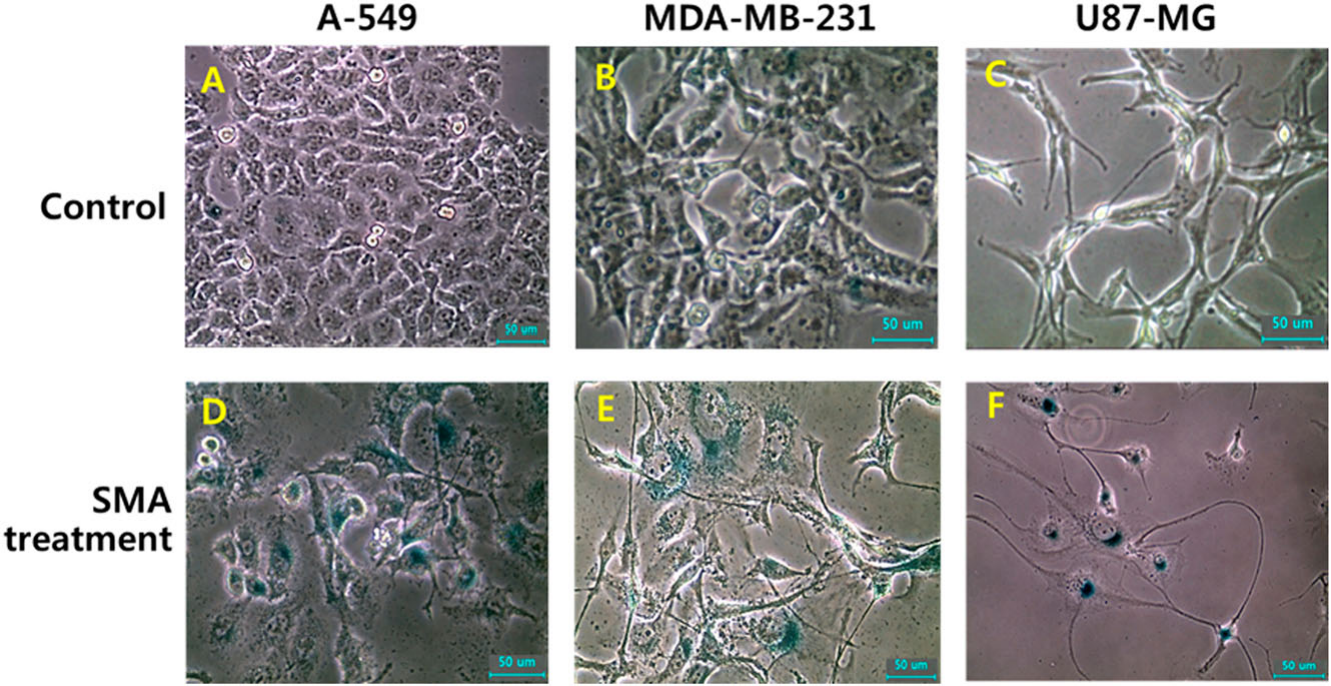
Change in cell morphology and senescence-associated-β-galactosidase activity in untreated control (A, B and C) and 1 μM SMA-treated (D, E and F) A-549, MDA-MB-231 and U87-MG cancer cells A-549, MDA-MB-231 and U87-MG cancer cells up to 2 weeks (×100). Cell changes, such as enlarged and flattened size, and star-shape, were frequently observed by SMA treatment. And a higher activity of senescence-associated-β-galactosidase stained with blue color were observed in cells treated with SMA. Scale bars: 50 μm.

The enzymatic activity of β-galactosidase was investigated in A-549, MDA-MB-231 and U87-MG cancer cells treated with 1 μM SAM, as shown in Figure 4. Following SMA treatment for up to 2 weeks, the size of the cells was gradually flattened and enlarged, and the morphological alternation, such as star-shape, was frequently observed by SMA treatment. Moreover, the high frequency of cells with activity of senescence-associated-β-galactosidase was also exhibited in A-549, MDA-MB-231 and U87-MG cancer cells treated with 1 μM SMA, implying that the cells have undergone cellular senescence.

### Analysis of cellular apoptosis and differentiation

**Figure 5.**
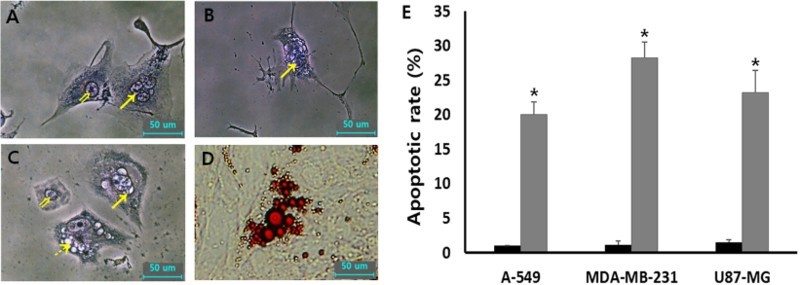
Morphological evaluation of cellular apoptosis and differentiation in 1 μM SMA-treated A-549 (A), U87-MG (B) and MDA-MB-231 (C and D) cancer cells. The fragmented nuclei (arrow) displayed in apoptotic cells were observed by Wright-Giemsa staining, compared with normal (double arrow) nuclei. Further, lipid droplets (dotted arrow)-like morphological alternations under light microscope (C) were observed by 1 μM SMA treatment and the droplets were stained with Oil red O solution (D). (E) Frequency of apoptotic cells with DNA fragmentation in untreated control (▪) and 1 μM SMA-treated (▪) A-549, MDA-MB-231 and U87-MG cancer cells. Asterisks (*) indicates significant (*P* < .05) difference between untreated control and SMA-treated cell lines, respectively.

The cellular apoptosis was analyzed by DNA fragmentation using Wright-Giemsa staining assay in A-549, MDA-MB-231 and U87-MG cancer cells treated with 1 μM SMA, as shown in Figure 5. The cells with fragmented nucleus were considered as apoptotic cells. Over 300 cells were analyzed for DNA fragmentation. The frequency of apoptotic cells in the untreated three types of cancer cell lines was observed at an extremely rare level. However, the rate of apoptotic cells with DNA fragmentation was 20.1 ± 1.8%, 28.2 ± 2.3% and 24.2 ± 3.2% in A-549, MDA-MB-231 and U87-MG cancer cells after treating with 1 μM SMA, respectively. A significant (*P* < .05) high rate of cellular apoptosis was induced by the treatment of SMA during prolonged culture time of up to 2 weeks. Moreover, the cells with lipid-like vesicles were frequently observed after SMA treatment and the vesicles reacted to Oil Red O solution commonly used for staining of neutral triglycerides and lipids, implying that the cells were differentiated into adipocytes.

### Activity of apoptosis and cancer-associated transcripts

**Figure 6.**
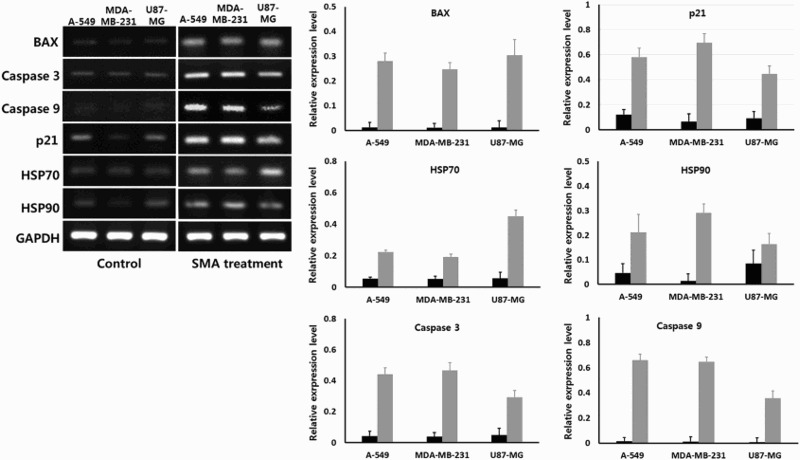
Expression level of transcripts related with intrinsic apoptosis and stress by real-time RT-PCR in untreated control (▪) and 1 μM SMA-treated (▪) A-549, MDA-MB-231 and U87-MG cancer cells. Asterisks (*) indicates significant (*P* < .05) difference between untreated control and SMA-treated cell lines, respectively.

The expression level of intrinsic apoptosis (BAX, caspase 3 and caspase 9) and stress (p21, HSP 70 and HSP90)-related transcripts was quantified by analysis of Ct value using real-time RT-PCR assay in A-549, MDA-MB-231 and U87-MG cancer cells treated with 1 μM SMA, as shown in Figure 6. The GAPDH was used as reference gene and its expression level was not significantly (*P* < .05) different among the untreated control and SMA-treated cancer cell lines examined. The intrinsic apoptosis-related transcripts (BAX, caspase 3 and caspase 9) and stress-related transcripts (p21, HSP70 and HSP90) were broadly detected at a low level in untreated control A-549, MDA-MB-231 and U87-MG cancer cells. However, the expression level of intrinsic apoptosis-related transcripts (BAX, caspase 3 and caspase 9) was significantly (*P* < .05) increased, and the expression level of stress-related transcripts (p21, HSP70 and HSP90) was also significantly (*P* < .05) increased in the A-549, MDA-MB-231 and U87-MG cancer cells treated with 1 μM SMA for up to 2 weeks.

## Discussion

The present study assessed the cytotoxic effects on the shortening of telomere length and cellular apoptosis by treatment of 1 μM SMA during prolonged culture time for up to 2 weeks in the various types of human cancer cell lines (A-549, MDA-MB-231 and U87-MG). In the present results, the treatment of SMA has induced shortening of telomere length by downregulating telomerase activity and decreasing the expression of telomerase-related transcripts (TERT and TERC). Furthermore, the high incidence of cells with senescence-associated β-glucosidase activity and cellular apoptosis was demonstrated by DNA fragmentation observed in the SMA-treated cancer cell lines. The increased expression of intrinsic apoptosis-related (BAX, caspase 3 and caspase 9) and stress-related (p21, HSP70 and HSP90) transcripts was further observed by SMA treatment. Morphological alternations, such as enlarged and star-shaped cells and cellular differentiation into adipocytes, were also observed in the cancer cell lines treated with SMA.

Arsenic compound is a commonly existing element that can be found in water, soil, rocks or organism, such as plants and animals, and the element is also released in the industrial processes. The two types of arsenic, including inorganic arsenite combined with other elements rather than carbon, and the organic arsenic combined with mainly carbon elements, exist in nature. However, a number of their compounds containing arsenic component are easily accumulated in the cells and tissues. It has been reported that the arsenic compounds are used for the induction of aneuploidy chromosomes by disruption of microtubule assembly and spindle formation (Ramírez et al. 1997), and DNA damage and cellular apoptosis by remarkable accumulation of ROS (Lynn et al. 2000; Schwerdtle et al. 2003). Additionally, certain arsenic compounds were also reported to decrease the expression of genes associated with DNA repair, such as p53 and damage-specific DNA-binding protein 2 (Hamadeh et al. 2002). Therefore, the inorganic and organic compounds containing arsenic are widely known as a cytotoxic inducing material; especially the inorganic arsenite and arsenate are more toxic than the organic arsenite compounds, as mutagenic agents against DNA, protein and other metabolites (Moore et al. 1997).

The rate of cell proliferation was remarkably inhibited by the treatment of arsenic compounds in various types of human cancer cell lines (Phatak et al. 2008; Ferrario et al. 2009; Ruiz-Ramos et al. 2009; Jeon et al. 2011a). Thus, the cytotoxic effects on the inhibition of cell proliferation by treatment of SMA have been continually attempted as a potential anti-tumor drug. Interestingly, our previous study has demonstrated that the IC_50_ values against SMA in the various cancer cell lines are highly lower than those of normal and adult stem cell lines, and it has thus been suggested that treatment of SMA in the cancer cell lines could be used for effective and successful chemotherapeutic drug at a proper concentration (Jeon et al. 2011a). Other studies have also shown that SMA is usually used for the treatment or alternative therapy for acute promyelocytic leukemia, prostate cancer cells and other cell lines (Shen et al. 1997; Soignet et al. 1998; Maeda et al. 2001). In our earlier investigation, the IC_50_ value against SMA was approximately 3 μM in the A-549, MDA-MB-231 and U87-MG cancer cell lines. In the present results, A-549, MDA-MB-231 and U87-MG cancer cell lines were prolongedly exposed to lower SMA concentration than IC_50_ values suggested in our previous study. And the SMA treatment at 1 μM concentration for up to 2 weeks was observed to increase doubling time of populations in A-549, MDA-MB-231 and U87-MG cancer cells, suggesting that the rate of cell proliferation of the cancer cells treated with SMA is efficiently inhibited by cytotoxic effects. Furthermore, our previous report has shown that the IC_50_ values are ∼30 and 15 μM SMA concentration in the adult stem cells and normal muscle cells derived from human dental tissues, respectively. Even though the present study has not investigated the cytotoxic effects, such as the proliferation & apoptosis in normal cell lines after prolonged exposure at 1 μM SMA concentration, we have assumed that the less cellular cytotoxic effects on cell proliferation by treatment of SMA are exhibited in the normal cell lines than those of cancer cell lines when compared with our earlier results, and probably that SMA could be considered as a selective anti-tumor drug for chemotherapy treatment. The selective cytotoxicity-inducing compounds could be more effective as anti-tumor drugs for therapeutic treatment of cancer cells with less cytotoxic effects in the normal cell lines than cancer cells (Jeon et al. 2011a; Moon et al. 2015).

In mammals, telomeres, a region of repetitive 5′-(TTGGGG)n-3′ sequences, are the components of each linear DNA ends. A DNA polymerase is responsible for the synthesis of new DNA stands; however, DNA synthesis is incomplete to replicate the 3′ end of each linear DNAs during cell division by their functional deficiency, called end-replication problem (Aubert and Lansdrop 2008; Arnoult and Karlseder 2015). Thus, the telomeric repeats in most of the normal cells are gradually shortened along with progressive cell division, namely replicative senescence. The erosion of DNA by fully shortening telomeric repeats in cell subsequently results in cellular senescence or crisis, and thus most of the differentiated normal cells display an arrest of cell cycle after 30–50 cell divisions (Allsopp et al. 1992; Levy et al. 1992; Ramírez et al. 2003; Jeon et al. 2011c). The stabilization or elongation of the telomeric repeats occurs through the activity of telomerase enzyme, an RNA-dependent DNA polymerase, which adds telomeric TTAGGG repeats to the end of each of G-quadruplex DNA strands (Aubert and Lansdrop 2008; Arnoult and Karlseder 2015). Even though most of the cancer or transformed cells possess short telomere repeats reached at almost senescent or crisis state, their telomeric repeats maintain a high level of telomerase activity. The embryonic stem cells and primordial germ cells, like cancer or transformed cells, also possess the high level of telomerase activity for self-renewal of the cells. These types of cells thus escape from replicative senescence and shortening telomeric repeats (Batista et al. 2011). The highly upregulated telomerase activity plays a very important role in the progression of malignant tumor cells for unlimited cell proliferation and is also one of the main biomarkers for cancer properties and can be distinguished from normal cells. In these points of view, several compounds inducing telomere shortening, downregulation of telomerase activity or their malfunction, such as G-quadruplex-interactive molecule BRACO-19, TMPyP4, RHPS4, telomestatin, GRN163L and others have been developed and attempted as anti-cancer drugs for chemotherapy treatment in various types of cancer cells (Burger et al. 2005; Salvati et al. 2007; Phatak et al. 2008; Röth et al. 2010; Fujimori et al. 2011; Woo et al. 2014). Meanwhile, a previous study has also suggested that SMA can integrate with telomeric repeats and could induce erosion or shortening of telomeric repeats, and thus can probably be considered as a potential anti-cancer drug for targeting telomeric repeats (Phatak et al. 2008). Especially, this compound selectively combines with relatively short telomere such as cancer cells than those of normal cells with relatively long telomere.

The present results clearly show that telomere shortening is induced in A-549, MDA-MB-231 and U87-MG cancer cells treated with SMA during prolonged cell culture time for up to 2 weeks. It has been shown in our previous report that any alternations in telomere repeats are not observed in the cancer cells treated with SMA at IC_50_ values for 48 h. The replicative senescence by telomere shortening is induced with successive cellular division under low levels of telomerase activity, as seen in fully differentiated cells. It has been reported that the rate of shortening telomere is approximately 48 bp per PDT in in vitro cultured fibroblasts (Allsopp et al. 1992; Levy et al. 1992). The treatment of SMA at a higher concentration during short culture time is probably thought to be a consequence of the cellular senescence and apoptosis by its cytotoxicity before replication senescence for telomere shortening. Moreover, the downregulation of telomerase activity was displayed by treatment of SMA in the present results. Our previous study has also demonstrated that telomerase activity is remarkably decreased in the cancer cells treated with SMA. It has been well proven that the upregulation of telomerase activity is associated with high expression level of TERT catalytic subunit, and TERC (Cao et al. 2003). The decreased expression of TERT and TERC resulted in the downregulation of telomerase activity. It has been reported that the treatment of SMA results in the downregulation of TERT transcript in leukemia cells (Glienke et al. 2006). The present study has demonstrated that the treatment of SMA results in highly decreased expression level of TERT and TERC transcripts, and subsequent downregulation of telomerase activity. As mentioned above, telomere shortening could be induced in the cancer cells through downregulating the expression level of telomerase activity along with the decrease in TERT and TERC transcripts’ level.

The β-galactosidase enzyme is a glycoside hydrolase that break glycosidic bonds. The endogenous lysosomal β-galactosidase protein is generally overexpressed in the senescent cells, and thus cellular aging and senescence is measured by its activity (Lee et al. 2006; Moon et al. 2015). The treatment of SMA has shown higher activity of senescence-associated-β-galactosidase in the present study, implying that cellular senescence was proceeding. It has been demonstrated that the most of the cells that reached cellular senescence were arrested at the G0/G1 phase of cell the cycle (Funayama and Ishikawa 2007). In other studies, the arrest of the cell cycle at the G1/S phase was induced by SMA treatment in various types of human cells (Ouyang et al. 2005; Kurokawa et al. 2014). Even though the change in cell cycle has not been investigated in the present study, we presumed that the cell population at the G1/S phase is probably increased by the treatment of SMA. Furthermore, it has been reported that the most of the cells that reached cellular senescence undergo morphological alterations with enlarged, flattened and irregular cell shape (Funayama and Ishikawa 2007; Moon et al. 2015). Our results have also shown morphological alternations into enlarged and star-shaped cells by SMA treatment, and the treated cells were probably undergoing the senescent status. The tumor and cancer cells also possess various degrees of differentiation capacity into multi-lineage cell types (Reya et al. 2016). It has further been pointed out that the morphological alterations, such as starry and flattened shape by the assembly of actin filament, are caused by cellular differentiation along with tumorous loss of the cancer cells (Pawlak and Helfman 2001). The morphological alternations of the cells treated with SMA might be a result of cellular senescence as well as cell differentiation. In a recent investigation, a lot of small vesicles were occasionally observed in the cytoplasm of the cells treated with SMA. And the vesicles were reacted with Oil Red O solution used for staining of lipid molecules, such as neutral triglycerides and the cells with vesicles were unquestionably differentiated into adipocytes after treatment of SMA. The available investigations on the cell differentiation into adipocytes by treatment of SMA are not still reported in the human cancer cell lines. However, we assumed that the cellular differentiation was apparently related with loss of tumorous characterizations in the A-549, MDA-MB-231 and U87-MG cancer cells treated with SMA.

The DNA fragmentation of the cells, during apoptosis, is a key step by the activation of endogenous endonucleases (Elmore 2007). A resistance to cellular apoptosis is considered as one of the typical characteristics of malignant cancer cells and the various methods for inducing cellular apoptosis are being continually developed and tried in cancer treatment (Elmore 2007; Fernald and Kurokawa 2013). In the present results, the A-549, MDA-MB-231 and U87-MG cancer cells were exposed to a 1 μM SMA concentration during prolonged culture time for up to 2 weeks, and the treated cells displayed a high frequency of DNA fragmentation by Wright-Giemsa staining, having high level of senescence-associated-β-galactosidase activity. Previous studies have demonstrated the high rate of apoptosis induced by the treatment of SMA in several types of human cancer cells (Bode and Dong 2000; Roboz et al. 2000; Glienke et al. 2006). Furthermore, we demonstrated that apoptosis-related transcripts are highly increased in the cancer cell lines treated with SMA. The extrinsic or intrinsic pathway causing cellular apoptosis by the treatment of SMA is still the question under debate. The BAX, caspase 3 and caspase 9 protein are generally related with the intrinsic apoptotic pathway (Dewson and Kluck 2009). In several other studies, it has also been reported that altering Bcl-2 family proteins and activating the intrinsic pathway are induced for apoptosis in human mesenchymal stem cells (Yadav et al. 2010) and cytotoxic effect through the activation of the intrinsic caspase-dependent apoptotic pathway is displayed in the human epithelial carcinoma cell line treated with arsenite (Arshad et al. 2015). Besides, we have demonstrated that the increased stress-related proteins, such as p21, HSP 70 and HSP 90, are also observed by SMA treatment. The expression level of p21 was increased by arsenite treatment in the human epithelial carcinoma cells (Arshad et al. 2015). And the increased p21 protein also induced the intrinsic apoptosis pathway and arrest of cell cycle (Cao et al. 2003). Therefore, the cellular apoptosis by SMA treatment may be due to the consequence of intrinsic apoptotic pathway of the cell. Meanwhile, the cellular apoptosis by arsenite was induced by activation of c-Jun N-terminal protein kinase and p38 mitogen-activated protein kinase pathways, key regulators of apoptosis (Hossain et al. 2000; Namgung and Xia 2000). It has further been reported that approximately 35% of HeLa S3 cells are found to be arrested in their cell cycle upon arsenite treatment and the cells subsequently enter cellular apoptosis, implying that the retardation or arrest of the cell cycle is one cause for apoptotic induction (Huang et al. 2000). As mentioned above, the increased PDT of the cancer cell lines by SMA treatment might be induced due to the retardation of the cell cycle and subsequent cellular apoptosis.

The cells with shortening of telomere repeats during progressive cell divisions are also induced to instability and fusion of the DNA strands, and telomere shortening is one of the possibilities for apoptosis (Capper et al. 2007). The mean telomere length for inducing cellular apoptosis by the instability and fusion of the DNA strands is approximately 12.8 telomeric repeats, six base pairs (Capper et al. 2007; Aubert and Lansdrop 2008; Arnoult and Karlseder 2015). Further, it has been suggested that telomere length of normal human cells at senescence is from 7 to 4 kbp (Karlseder et al. 2002). In another report, the rate of cellular apoptosis was proportionally increased by gradual shortening of telomeric repeats in HL60 cells treated with an anti-cancer drug; however, the length of telomeric repeats at a high rate of apoptotic cells was approximately 18 kbp (Ramírez et al. 2003). The variation in telomere length inducing apoptosis in each study may be due to various methods and type of restriction enzyme used for the analysis of telomere length. In the present study, the telomere length was 3.9, 4.5 and 2.6 kbp in the A-549, MDA-MB-231 and U87-MG cancer cells treated with 1 μM SMA, respectively. The cause of apoptosis in the present study was unclear, whether it is due to the shortened telomere or other signaling pathways, including the intrinsic apoptosis pathway. Even though our results have shown telomere shortening and downregulated telomerase activity in the cancer cells treated with SMA, cellular apoptosis might also be induced by another apoptotic signaling pathway, such as arrest in the cell cycle, DNA damage and others rather than telomere shortening. It has further been reported that increased activation of telomeric repeat-binding factor 2 (TRF2) is inhibited due to the induction of cellular senescence in the cells having critically short telomeres, implying that cellular senescence and apoptosis are controlled by different factor(s), such as TRF2 protein, in the cells with fully shortened telomeres, rather than by a complete shortening of telomeric repeats (Karlseder et al. 2002).

In conclusion, the present study has shown that the SMA compound evidently exhibits cellular cytotoxicity by the induction of telomere shortening and apoptosis in three types of cancer cell lines, including the human A-549 lung adenocarcinoma, MDA-MB-231 breast adenocarcinoma and U87-MG brain glioblastoma astrocytoma. Our previous results have also shown that the normal cells, such as muscle cells and adult mesenchymal cells derived from human dental tissues, possess extremely higher IC_50_ value than those of cancer cell lines. In the present study, the cancer cells treated with SMA could be induced to cellular cytotoxicity, such as induction of telomere shortening and apoptosis, and normal cells treated at a proper treatment concentration could exhibit less cellular cytotoxicity with prolonged dose period. Thus, the SMA might be a potential and effective chemotherapy agent or alternative treatment for cancer cells. However, the effects of SMA are to be carefully investigated in in vivo treatment and different types of cancer and normal cell lines.
